# Editorial Notes

**Published:** 1893-11

**Authors:** 


Dr. Richard J. Trippe, the surgeon of Chattanooga, Tenn., was married on the 8th inst. to Miss Nellie Fewell, of Meridan, Miss. The RECORD wishes them a long and happy life.

---

THE RECORD was favored with a pleasant call from Dr. A. W. Griggs, of West Point, Ga., a few days ago.

